# Quantitative evaluation of SARS-CoV-2 inactivation using a deep ultraviolet light-emitting diode

**DOI:** 10.1038/s41598-021-84592-0

**Published:** 2021-03-03

**Authors:** Takeo Minamikawa, Takaaki Koma, Akihiro Suzuki, Takahiko Mizuno, Kentaro Nagamatsu, Hideki Arimochi, Koichiro Tsuchiya, Kaoru Matsuoka, Takeshi Yasui, Koji Yasutomo, Masako Nomaguchi

**Affiliations:** 1 Department of Post-LED Photonics Research, Institute of Post-LED Photonics, Tokushima University, 2-1 Minami-Josanjima, Tokushima, Tokushima 770-8506 Japan; 2 Department of Mechanical Science, Graduate School of Technology, Industrial and Social Sciences, Tokushima University, 2-1 Minami-Josanjima, Tokushima, Tokushima 770-8506 Japan; 3 PRESTO, Japan Science and Technology Agency (JST), 2-1 Minami-Josanjima, Tokushima, Tokushima 770-8506 Japan; 4 Research Cluster on “Multi-Scale Vibrational Microscopy for Comprehensive Diagnosis and Treatment of Cancer”, Tokushima University, 2-1 Minami-Josanjima, Tokushima, Tokushima 770-8506 Japan; 5 Department of Microbiology, Graduate School of Biomedical Sciences, Tokushima University, 3-18-15 Kuramoto, Tokushima, Tokushima 770-8503 Japan; 6 Department of Electrical and Electronic Engineering, Graduate School of Technology, Industrial and Social Sciences, Tokushima University, 2-1 Minami-Josanjima, Tokushima, Tokushima 770-8506 Japan; 7 Department of Immunology and Parasitology, Graduate School of Biomedical Sciences, Tokushima University, 3-18-15 Kuramoto, Tokushima, Tokushima 770-8503 Japan; 8 Department of Medical Pharmacology, Graduate School of Biomedical Sciences, Tokushima University, 3-18-15 Kuramoto, Tokushima, Tokushima 770-8503 Japan; 9 Department of Interdisciplinary Researches for Medicine and Photonics, Institute of Post-LED Photonics, Tokushima University, 3-18-15 Kuramoto, Tokushima, Tokushima 770-8503 Japan; 10 Research Cluster On “Immunological Diseases”, Tokushima University, 3-18-15 Kuramoto, Tokushima, Tokushima 770-8503 Japan

**Keywords:** SARS-CoV-2, Inorganic LEDs, Biophotonics, Viral infection

## Abstract

Inactivation technology for severe acute respiratory syndrome coronavirus 2 (SARS-CoV-2) is certainly a critical measure to mitigate the spread of coronavirus disease 2019 (COVID-19). A deep ultraviolet light-emitting diode (DUV-LED) would be a promising candidate to inactivate SARS-CoV-2, based on the well-known antiviral effects of DUV on microorganisms and viruses. However, due to variations in the inactivation effects across different viruses, quantitative evaluations of the inactivation profile of SARS-CoV-2 by DUV-LED irradiation need to be performed. In the present study, we quantify the irradiation dose of DUV-LED necessary to inactivate SARS-CoV-2. For this purpose, we determined the culture media suitable for the irradiation of SARS-CoV-2 and optimized the irradiation apparatus using commercially available DUV-LEDs that operate at a center wavelength of 265, 280, or 300 nm. Under these conditions, we successfully analyzed the relationship between SARS-CoV-2 infectivity and the irradiation dose of the DUV-LEDs at each wavelength without irrelevant biological effects. In conclusion, total doses of 1.8 mJ/cm^2^ for 265 nm, 3.0 mJ/cm^2^ for 280 nm, and 23 mJ/cm^2^ for 300 nm are required to inactivate 99.9% of SARS-CoV-2. Our results provide quantitative antiviral effects of DUV irradiation on SARS-CoV-2, serving as basic knowledge of inactivation technologies against SARS-CoV-2.

## Introduction

Severe acute respiratory syndrome coronavirus 2 (SARS-CoV-2), causing coronavirus disease 2019 (COVID-19), emerged in late 2019 and spread globally to become a pandemic^1,2^. The COVID-19 pandemic has caused severe damage to public health and economics worldwide. Although several preventive measures against SARS-CoV-2, such as containment, social distancing, wearing face masks, and washing hands, have been extensively taken, people infected with SARS-CoV-2 and resultant deaths by COVID-19 are still increasing^3–5^. Thus, to decrease the infection risk and to restore society and the economy to how they were before the onset of the disease, more direct and effective preventive means against SARS-CoV-2 are required.

There are several approaches to mechanically inactivate SARS-CoV-2, e.g., chemical, thermal, and photochemical methods. Chemicals, such as alcohol-based disinfectants, are frequently and widely used to inactivate SARS-CoV-2 on hands and the surfaces of equipment^6,7^. Thermal treatment is also known to be effective for SARS-CoV-2 inactivation^8,9^. Photochemical inactivation, especially using deep ultraviolet (DUV) light (λ < 300 nm), is the most time-saving and efficient way to inactivate SARS-CoV-2 and related coronaviruses^10–18^. DUV light irradiation exerts a critical effect on the infectivity of microorganisms and viruses probably by damaging their RNAs^19^. DUV light can inactivate viruses in various environments with minimal undesirable effects on the target materials. Among DUV light sources used for the photochemical inactivation method, a DUV light-emitting diode (DUV-LED) is much more attractive because of its small size and low toxicity compared to mercury lamps that are limited in usage in accordance with the Minamata Convention on Mercury adopted by the United Nations Environment Programme in 2013^20,21^. Researchers have provided proof-of-concept studies on the DUV-LED inactivation of SARS-CoV-2^14,18^. However, quantitative analyses on SARS-CoV-2 inactivation by DUV-LED irradiation at various wavelengths need to be carried out.

In the present study, we quantified the inactivation efficacy of DUV-LED on SARS-CoV-2. To quantitatively determine the effective doses of DUV-LED light on SARS-CoV-2, we defined the culture medium of SARS-CoV-2 suitable for DUV-LED irradiation and optimized the DUV-LED irradiation apparatus. Under these conditions, we evaluated the relationship of SARS-CoV-2 infectivity and the irradiation dose of DUV-LED light at three major wavelengths (265, 280, and 300 nm). The results obtained would provide a common standard for SARS-CoV-2 inactivation useful for the development of DUV irradiation apparatuses and techniques.

## Results

### Media conditions for DUV-LED irradiation of SARS-CoV-2

**Figure 1:**
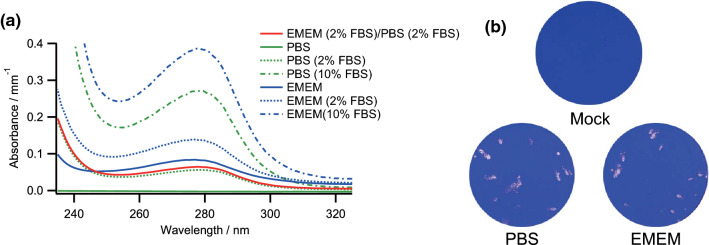
Effects of the culture media on light absorbance and SARS-CoV-2 infectivity. (**a**) Absorbance spectra of EMEM, FBS, PBS, and their mixed media. The absorbance by the various media indicated was monitored using a UV–visible spectrometer. FBS concentrations in the culture media are shown in parentheses. EMEM (2% FBS)/PBS (2% FBS), red line; EMEM containing 2% FBS diluted with PBS containing 2% FBS by tenfold. (**b**) Effects of the culture media on viral infectivity. Virus samples were diluted with EMEM containing 2% FBS (EMEM) or PBS containing 2% FBS (PBS). The samples appropriately diluted for plaque assays were inoculated into Vero E6 cells. On day 3 post-infection, cells were fixed and stained to visualize plaques. Representative data from six independent assays are shown.

Viruses are generally stocked in a culture medium composed of balanced salts, glucose, amino acids, vitamins, serum, and antibiotics to maintain their viability and infectivity. The chemicals, especially proteins and amino acids, in ordinal culture media absorb DUV light well^22–24^. Thus, for quantitative evaluations of the DUV-LED irradiation effects on SARS-CoV-2, we addressed to minimize DUV-LED light absorbance by the culture media during irradiation as follows. To determine the culture medium of SARS-CoV-2 suitable for DUV-LED irradiation, we examined the absorbance spectra of Eagle's minimal essential medium containing 2 mM L-glutamine and antibiotics (EMEM) and fetal bovine serum (FBS), which are used for virus propagation, and phosphate buffered saline (PBS). The absorbance spectra of EMEM, FBS, PBS, and their mixed media were monitored using a UV–visible spectrometer (240–320 nm). As shown in Fig. 1a, all culture media, except for PBS without FBS, exhibited an absorption peak around 280 nm, which is due to proteins or amino acids in EMEM and FBS^22–24^. The absorbance by PBS without FBS, which contains NaCl, KCl, and sodium phosphate, was much lower than 0.01 mm^−1^ at all wavelengths (265, 280, and 300 nm). The difference in the absorbance between PBS and EMEM was 0.073 mm^−1^ for 265 nm, 0.084 mm^−1^ for 280 nm, and 0.035 mm^−1^ for 300 nm. The absorbance by both PBS and EMEM increased in an FBS-concentration dependent manner. The increase in absorbance by the addition of 2% FBS to EMEM and PBS was about 0.042 mm^−1^ for 265 nm, 0.054 mm^−1^ for 280 nm, and 0.01 mm^−1^ for 300 nm. The absorbance by PBS containing 2% FBS was lower than that by EMEM without FBS.

The results, as shown in Fig. 1a, indicated that the culture media suitable for DUV-LED irradiation should contain a minimum amount of EMEM and FBS to significantly reduce the light absorbance. Since we used EMEM containing 2% FBS as a virus stock medium, we diluted the virus with PBS containing 2% FBS by tenfold to decrease the absorbance between 240 and 320 nm. The absorbance by the diluted medium decreased to a similar level of the absorbance by PBS containing 2% FBS at all the wavelengths of 265, 280, and 300 nm (note the red and green dashed lines in Fig. 1a). The absorbance by the diluted medium was estimated to be 0.052 mm^−1^ for 265 nm, 0.064 mm^−1^ for 280 nm, and 0.015 mm^−1^ for 300 nm (Fig. 1a). To confirm applicability, we determined SARS-CoV-2 infectivity in the diluted media by plaque assays, as shown in Fig. 1b. SARS-CoV-2 infectivity in the diluted medium (PBS containing 2% FBS) was similar to that in the virus medium diluted with EMEM containing 2% FBS (1.4 × 10^4^ plaque forming unit (PFU)/mL for PBS containing 2% FBS and 1.2 × 10^4^ PFU/mL for EMEM containing 2% FBS on average). We thus used PBS containing 2% FBS as diluents to prepare the virus samples for DUV-LED irradiation.

### Development of DUV-LED irradiation apparatus

**Figure 2:**
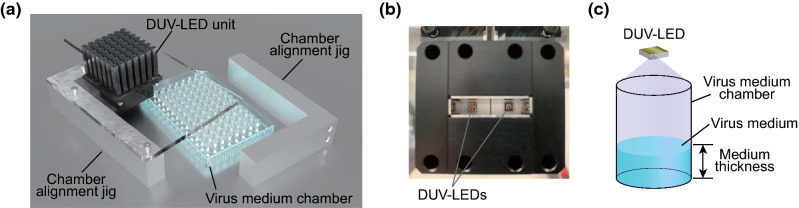
Schematics of the DUV-LED irradiation apparatus. (**a**) Three-dimensional view of the DUV-LED irradiation apparatus. The virus medium chamber is set just under the DUV-LED unit and is supported by the chamber alignment jigs. (**b**) Bottom view of the DUV-LED unit with two DUV-LEDs. (**c**) Schema of the virus medium chamber used for DUV-LED irradiation. The DUV-LED irradiation area is large enough to obtain uniform irradiation.

We developed an irradiation apparatus to quantitatively analyze SARS-CoV-2 inactivation by DUV-LED. As shown in Fig. 2a, the DUV-LED irradiation apparatus is composed of a DUV-LED unit, a 96-well plate as a virus medium chamber, and chamber alignment jigs. The DUV-LED unit consists of two DUV-LED chips and a heat sink (Fig. 2b). Upon irradiation, the virus medium chamber was set just under the DUV-LED unit. Virus inoculum was placed in a defined well of the chamber and was irradiated by the DUV-LED from the top. The DUV-LED irradiation area was set large enough to obtain uniform irradiation (Fig. 2c). For correct irradiation, the virus medium chamber is fixed by chamber alignment jigs at both sides (Fig. 2a). This apparatus was constructed for the DUV-LEDs operating at a center wavelength of 265, 280, and 300 nm.

To determine the effective power densities, we first monitored the irradiation power densities at each wavelength irradiating on the well of the chamber. The measured values at the bottom of the well were 134 µW/cm^2^ for 265 nm, 131 µW/cm^2^ for 280 nm, and 1.03 mW/cm^2^ for 300 nm. In this study, since we used 100 µL of the virus medium in a chamber, the thickness of the virus medium was calculated to be 3.1 mm. Based on the results described in the previous section (Fig. 1), the transmittances through the culture medium (3.1 mm thickness) were estimated to be 69.0% for 265 nm, 63.3% for 280 nm, and 89.8% for 300 nm. Thus, the effective power densities of the DUV-LEDs, the actual power densities utilized for virus inactivation, were calculated to be 92 µW/cm^2^ for 265 nm, 83 µW/cm^2^ for 280 nm, and 925 µW/cm^2^ for 300 nm. In this study, we utilized the effective power densities to evaluate the inactivation effect of the DUV-LEDs.

### Inactivation of SARS-CoV-2 using a DUV-LED at various wavelengths

**Figure 3:**
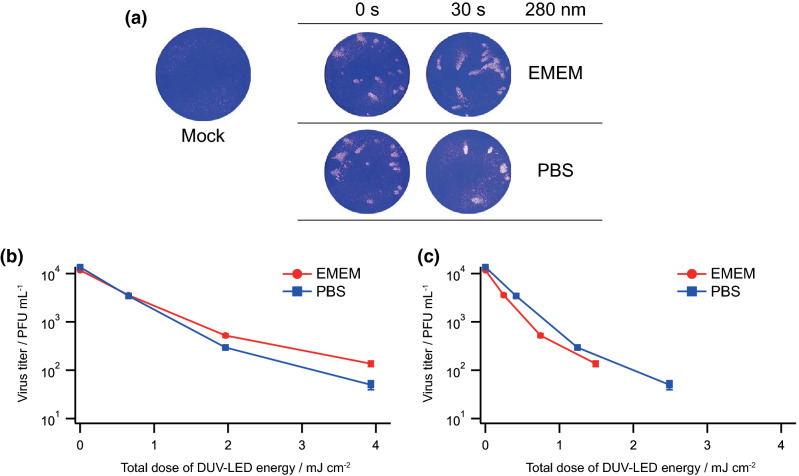
Effects of culture media on the inactivation efficacy of SARS-CoV-2 at 280 nm DUV. (**a**) Plaque assays. Virus stocks were diluted with EMEM containing 2% FBS (EMEM) or PBS containing 2% FBS (PBS). The diluted samples were exposed to DUV-LED at 280 nm wavelength for various irradiation time periods (5, 15, and 30 s). The irradiated samples were appropriately diluted, if necessary, for plaque assays as described in Fig. 1b. Representative data (irradiation for 0 and 30 s) from six independent assays are shown. (**b** and **c**) Inactivation efficacy at various total doses of DUV-LED energy with (**c**) and without (**b**) normalization by transmittances. The transmittances of the culture media of the virus stock diluted with EMEM containing 2% FBS and that with PBS containing 2% FBS at 280 nm were 37.9% and 63.3%, respectively. Mean values (M) ± standard errors (SE) are shown (N = 6).

Since culture media absorb DUV light at 280 nm most efficiently (Fig. 1a), we examined how the culture media would affect the viral inactivation efficacy using a DUV-LED at this wavelength. The virus stock was diluted by tenfold with EMEM containing 2% FBS or PBS containing 2% FBS, placed in a defined well of a 96-well plate (chamber), and irradiated using a DUV-LED at 280 nm wavelength. Virus infectivity was determined using plaque assays with Vero E6 cells. As shown in Fig. 3a, the plaque numbers for samples diluted with PBS containing 2% FBS were significantly reduced relative to those for EMEM containing 2% FBS. The total dose of DUV-LED energy required to inactivate SARS-CoV-2 at each assay point was lower for samples diluted with PBS containing 2% FBS than those for EMEM containing 2% FBS (Fig. 3b). This demonstrated that the low light absorbance by the media resulted in the high inactivation efficacy by DUV-LED. Because the light absorbance by EMEM containing 2% FBS is higher than that by PBS containing 2% FBS (Fig. 1a), the total dose of DUV-LED energy was recalculated based on their transmittances (Fig. 3c). The recalculated values would represent the actual DUV-LED energy imposed on the viruses. We found that virus samples in EMEM containing 2% FBS were more readily inactivated than those in PBS containing 2% FBS at the same DUV-LED energy level. This is probably due to indirect DUV effects caused by EMEM containing 2% FBS for irradiation. Thus, in order to accurately quantify the effects of DUV-LED irradiation, the inactivation efficacy needs to be determined using the culture media with the lowest absorbance.

**Figure 4:**
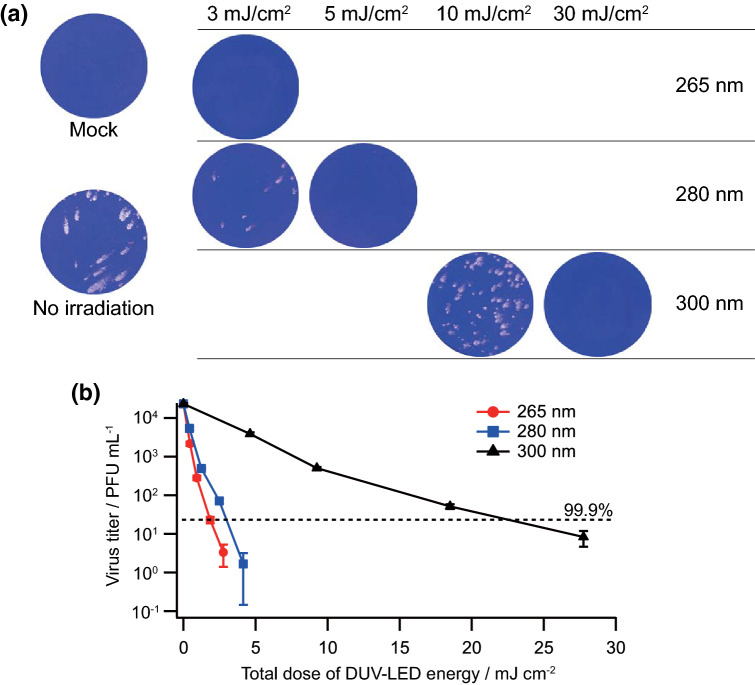
Effects of DUV-LEDs on SARS-CoV-2 inactivation. (**a**) Plaque assays. Virus samples were diluted with PBS containing 2% FBS and exposed to DUV-LEDs for various irradiation time periods. The irradiated samples were appropriately diluted, if necessary, for plaque assays as described in Fig. 1b. Representative data from six independent assays are shown. (**b**) Inactivation efficacy at various total doses of DUV-LED energy. The black broken lines show 99.9% inactivation of virus infectivity as indicated. M ± SE are shown (N = 6).

We then monitored the inactivation effects of the DUV-LEDs (265, 280, and 300 nm) under the conditions suitable for irradiation described above (Figs. 1, 2, 3). The effective irradiation power densities were set at a constant value of 92 µW/cm^2^ for 265 nm, 83 µW/cm^2^ for 280 nm, and 925 µW/cm^2^ for 300 nm, and the total dose of DUV-LED energy was controlled by varying the exposure time. The virus samples were diluted by tenfold with PBS containing 2% FBS, exposed to the DUV-LEDs, and subjected to plaque assays (Fig. 4a). Virus inocula for the positive controls without DUV-LED irradiation exhibited 2.3 × 10^4^ PFU/mL of virus infectivity on average. The irradiation of the DUV-LEDs decreased plaque numbers in a wavelength- and dose-dependent manner. Based on the data of Fig. 4a, the SARS-CoV-2 inactivation curves by DUV-LED irradiation are graphically shown in Fig. 4b. At all wavelengths, the plaque numbers decreased exponentially with respect to the total dose of DUV-LED energy. The DUV-LED operating at a wavelength of 265 nm exhibited the highest inactivation effect on SARS-CoV-2. The initial inactivation coefficient, which was defined by Eq. 1 in the Methods section, was estimated to be 5.1 cm^2^/mJ for 265 nm, 3.6 cm^2^/mJ for 280 nm, and 0.39 cm^2^/mJ for 300 nm. We also evaluated the minimal requirement of the total dose of DUV-LED energy for SARS-CoV-2 inactivation. To achieve the 99.9% inactivation of SARS-CoV-2, the total doses of DUV-LED energy required were found to be 1.8 mJ/cm^2^ for 265 nm, 3.0 mJ/cm^2^ for 280 nm, and 23 mJ/cm^2^ for 300 nm. In summary, we have obtained quantitative data here regarding the wavelength, power density, and irradiation time required to effectively inactivate SARS-CoV-2. Our quantitative data could be utilized to estimate the SARS-CoV-2 inactivation efficacy in various environments and circumstances. That is, total dose of DUV irradiation used in this study is expected to be also effective for SARS-CoV-2 inactivation in the public utilities, such as doors, buses etc., since we calculated the actual dose of DUV irradiated to viruses. For such estimations, transmittances in individual environments are essential. While further experiments to validate SARS-CoV-2 inactivation in actual environments and conditions, where DUV-LED irradiation is used, are needed to be done, our data are critical to establish SARS-CoV-2 inactivation techniques and apparatuses.

### Effects of DUV-LED irradiation on radical species production in the culture medium

To exclude the possibility that the SARS-CoV-2 inactivation we observed (Fig. 4) is caused by indirect effects of DUV-LED irradiation, we examined the production of radical species in the culture medium. Since the photon energy is high in the DUV wavelength region, the irradiation of DUV-LED may produce unexpected chemical species by affecting chemicals, such as proteins and amino acids, in the culture medium^25^. One possible chemical species generated by the DUV-LEDs are radical species such as the superoxide anion radical and hydroxyl radical.

The generation of radical species by DUV-LED irradiation was evaluated by the electron spin resonance (ESR) method. We used 5, 5-dimethyl-1-pyrroline N-oxide (DMPO) as the trapping agent for the superoxide anion radical and hydroxyl radical^26^. The irradiation power densities and the exposure time of the DUV-LEDs were set at 0.94 mW/cm^2^ and 160 s, respectively, for all the wavelengths (265, 280, and 300 nm), and thus, the total dose of DUV-LED energy was 150 mJ/cm^2^ at all wavelengths.

**Figure 5:**
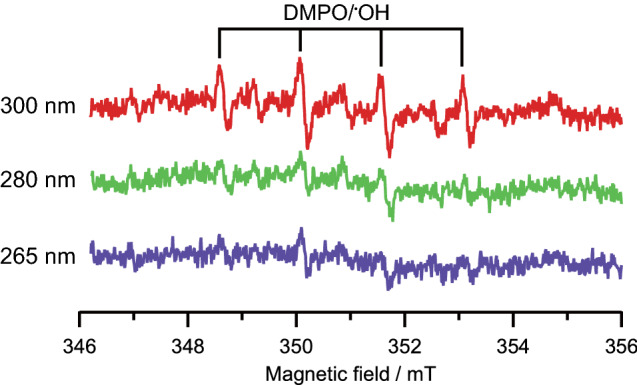
ESR spectra of culture media after DUV-LED irradiation. DMPO was used as the trapping agent of radical species. Details of this assay procedure are described in the Methods section.

The ESR spectra of the culture media after each DUV-LED irradiation are shown in Fig. 5. The spectral lines of the radical species trapped by DMPO were observed at all wavelengths, and they were identified as DMPO/^·^OH from the hyperfine coupling constants (a^N^ = a^H^ = 1.49 mT)^27^. The highest ESR spectral intensity was observed for DUV-LED irradiation at 300 nm, followed by that at 280 nm and 265 nm. Given that radial species affect the inactivation of SARS-CoV-2, the DUV-LED irradiation at 300 nm would have higher impacts on the viral infectivity than those at 280 nm and 265 nm. However, our results on SARS-CoV-2 inactivation (Fig. 4) were just the opposite. Taken together, radical species produced by DUV-LEDs irradiation are highly unlikely to affect SARS-CoV-2 inactivation.

## Discussion

In the current global pandemic of SARS-CoV-2, means to inactivate viruses and prevent infections must be taken. DUV irradiation is one powerful and effective way to combat various microorganisms including SARS-CoV-2 and related coronaviruses^10–18^. However, in order to establish commonly and widely usable inactivation apparatuses/techniques using DUV, reliable quantified data that can serve as a solid standard for their development are required; the length of the DUV irradiation periods, which wavelength, and what doses of DUV are needed for SARS-CoV-2 inactivation. In this study, we focused on the quantitative evaluation of SARS-CoV-2 inactivation by DUV-LEDs at three major wavelengths (265, 280, and 300 nm).

To this end, it is crucial to properly quantify the actual dose of DUV, i.e., measured values for irradiated power densities minus those for the light absorbance by the culture media. Transmittances can be calculated by the light absorption by media and the medium thickness in a chamber. The culture media (EMEM and FBS) used for virus stock preparations exhibited a high absorbance of DUV. To minimize this, we chose PBS containing 2% FBS as a diluent for the virus stocks. Irradiation apparatuses were designed to guarantee the uniform light intensity of DUV irradiation to the virus samples in a chamber. We monitored the irradiation power densities, calculated transmittances in a constant thickness of virus samples (3.1 mm) in a chamber, and finally estimated the actual dose of DUV energy imposed on the viruses. Indeed, the inactivation efficacy of SARS-CoV-2 was affected by the culture media used for DUV-LED irradiation. Different antiviral effects were noted for EMEM- and PBS-based media, underscoring the importance of the culture media used and the DUV energy dose based on their transmittances. Taken all together, we successfully obtained accurate and quantitative data on the relationship between SARS-CoV-2 inactivation and the total dose of DUV energy at each wavelength. These results provide pivotal information that should be considered to assess the inactivation of a variety of viruses by DUV-LEDs.

While the biological effects of DUV light have been widely proven across different types of viruses^14,16–19,28–32^, the precise mechanism by which the DUV light inactivates viruses remains to be elucidated^19^. Several studies reported that virus inactivation by DUV irradiation is primarily caused by the photochemical reactions of nucleic acids^19,33,34^. These reactions induce the formation of covalent-linked dimers via the fusion of two adjacent pyrimidines in RNAs. By these harmful nucleic acid alterations, viruses are inactivated. The other inactivation mechanisms by the deleterious effects of DUV on both RNAs and proteins, such as the RNA–protein cross-linking and site-specific damages to RNAs and proteins, also have been suggested^19,35,36^. Another important DUV-induced inactivation pathway is the generation of radical species, which can damage RNAs and proteins through oxidation^37,38^. Our results showed that DUV irradiation at 265 nm (absorption peak of RNA) most efficiently inactivates SARS-CoV-2, whereas radical production in the culture media is the highest at 300 nm DUV among the wavelengths tested (265, 280, and 300 nm). Under the conditions used in this study, it is reasonable to rule out the possible involvement of radical generations by DUV irradiation in causing the SARS-CoV-2 inactivation as a major determinant. The inactivation of SARS-CoV-2 by DUV-LED in our experiments may be due to the DUV-induced nucleic acid modification alone or in combination with other effects on viral proteins and/or lipid bilayers in the viral envelope. Needless to mention, the mechanism for SARS-CoV-2 inactivation by DUV remains to be determined. Elucidating the precise mechanism would lead to the improvement and generation of photochemical inactivation and/or combination (with chemicals and thermal treatments) techniques. In addition, recent studies showed that DUVs with shorter wavelengths at 222 nm and 254 nm inactivate SARS-CoV-2^15,16,39–41^. In view of virus inactivation, it is well known that 265 nm UV light is the adsorption peak of RNA, and that UV lights with shorter wavelengths exhibit greater photon energy. Thus, further studies, taking account of the inactivation efficacy, safety, effects on environments, and lifetime and cost of light sources, are necessary to develop a means to highly efficiently inactivate SARS-CoV-2.

In conclusion, we demonstrate here for the first time, to the best of our knowledge, the quantitative relationship between the SARS-CoV-2 inactivation efficacy and the actual dose of DUV-LED energy at major wavelengths (265, 280, and 300 nm). The comparative analysis using three distinct wavelengths of DUV-LED in the same conditions revealed that DUV at 265 nm wavelength most efficiently inactivates SARS-CoV-2, and that what dose of DUV at each wavelength can result in what extent of decrease in SARS-CoV-2 infectivity, i.e., the degree of virus inactivation. Using our quantitative data, one can estimate the SARS-CoV-2 inactivation efficacy in various environments, such as liquids, airflows, aerosols, and equipment surfaces. Transmittances in individual environments are essential for the estimation. Our findings provide a basic knowledge on the anti-SARS-CoV-2 technology, which is applicable to the other pathogenic viruses.

## Methods

### Cell and virus preparation

Vero (ATCC CCL-81) and Vero E6 (ATCC CRL-1586) cells were cultured and maintained in EMEM containing 10% heat-inactivated FBS (Biosera, Nuaille, France), 2 mM L-glutamine, and antibiotics (REF 10378-016, Thermo Fisher Scientific Inc., MA, USA). SARS-CoV-2 isolate (SARS-CoV-2/Hu/DP/Kng/19-020, Genbank: LC528232) was obtained from Kanagawa Prefectural Institute of Public Health, Chigasaki, Kanagawa, Japan. The virus was inoculated into Vero cells (80–90% confluency) in EMEM containing 2% FBS, 2 mM L-glutamine, and antibiotics. On day 2 post-infection, the culture supernatant was harvested and filtered through a 0.45 μm Minisart Syringe Filter (Sartorius Stedim Biotech GmbH, Goettingen, Germany) for virus stocks. Aliquots were stored at − 80 °C until use.

### Plaque assay

Vero E6 cells (2.0 × 10^5^ cells) were seeded on a 12-well plate and cultured overnight. Cells were infected with tenfold serial dilutions of a virus stock in EMEM containing 2% FBS and were incubated at 37 °C for 1 h. Inocula were replaced with 1 mL of a 0.5% suspension of methyl cellulose #4000 (REF 11675-82, Nacalai Tesque Inc., Kyoto, Japan) in EMEM containing 2% FBS, 2 mM L-glutamine, and antibiotics. Cells were incubated at 37 °C for 3 days, fixed, and stained with 10% formaldehyde (REF 068-03841, FUJIFILM Wako Pure Chemical Corporation, Osaka, Japan) containing 0.5% crystal violet (REF 031-04852, FUJIFILM Wako Pure Chemical Corporation, Osaka, Japan). The titer of the SARS-CoV-2 stocks was 1.3–2.6 × 10^5^ PFU/mL.

### DUV-LED irradiation

Just before DUV-LED irradiation, the virus stocks were appropriately diluted by EMEM containing 2% FBS (1.0 × 10^5^ PFU/mL), and then by PBS containing 2% FBS or EMEM containing 2% FBS to generate virus inoculum (1.0 × 10^4^ PFU/mL). For each irradiation experiment, 100 µL of virus inoculum was placed in a defined well (E4) of a 96-well plate, and the plate was exposed to the designated irradiation wavelength and time. After irradiation, each virus inoculum was subjected to the plaque assay.

### Absorbance spectroscopy

Absorbance spectra of samples in a quartz cell with 10-mm path length were measured using a UV–visible spectrometer (UV-1280, Shimadzu, Kyoto, Japan). Since the absorbance of EMEM containing 10% FBS and PBS containing 10% FBS were beyond the upper detection limit of the spectrometer, these media were diluted with distilled water, and the corresponding absorbance spectra were corrected according to the dilution ratios.

### DUV-LED irradiation apparatus

A home-built DUV-LED irradiation apparatus was used in this study. The DUV-LED irradiation apparatus consisted of a DUV-LED unit (CCS Inc., Kyoto, Japan), a virus medium chamber (REF3595, Corning Inc., NY, USA), and chamber alignment jigs made of aluminum alloy bars. The DUV-LED unit and a chamber alignment jig were fixed on an acrylic plate. The other chamber alignment jig was freely movable. The virus medium chamber was placed just under the DUV-LED unit and aligned by the fixed and freely movable jigs.

### Irradiation power density measurement

The irradiation power density of DUV-LED at each wavelength was obtained by measuring the transmitted light through a well of a virus medium chamber. We perforated a hole (the same diameter, 6.4 mm, with the bottom of the well) at the defined well (E4). The total power of the transmitted DUV-LED light was monitored by a power meter (detection size of 9.5 mm in diameter; S120VC, Thorlabs Inc., NJ, USA) that was set just under the hole. The irradiation power density was calculated by the total power of the transmitted light divided by the area of the well bottom.

### Inactivation coefficient

Because SARS-CoV-2 infectivity appeared to be exponentially reduced in inverse correlation with the total dose of DUV-LED energy, the dose–response curve can be fitted with the following equation:

1 $$N = N_{init} \exp \left( { - \alpha I_{total} } \right),$$

where *N*, *N*_init_, and *I*_total_ represent the number of plaques, the initial number of plaques, and the total dose of the DUV-LED energy, respectively. We defined the inactivation coefficient as *α* in Eq. (1).

### Electron spin resonance (ESR) spectroscopy

Radical species in the culture media generated by DUV irradiation were measured by ESR spectroscopy with the spin-trapping method^42,43^, and 5,5-dimethyl-1-pyrrorine-N-oxide (DMPO, Labotec Co., Ltd., Tokyo, Japan) was used as the spin trapping reagent. For ESR spectroscopy, 95 µL of EMEM containing 2% FBS, diluted with PBS containing 2% FBS by tenfold, was mixed with 5 µL of 100 mM DMPO. The mixed solution was put in a chamber of a 96-well plate and was irradiated by the DUV-LED irradiation apparatus developed in this study. The irradiation power densities and the exposure time of the DUV-LEDs were set at 0.94 mW/cm^2^ and 160 s, respectively, for all the wavelengths of 265, 280, and 300 nm; and thus, the total dose of DUV-LED energy was 150 mJ/cm^2^ at all wavelengths. After the DUV-LED irradiation, the mixed solution was immediately transferred to three sections of glass capillaries (10 µL, Drummond Co., Broomall, PA, USA) and set into the ESR spectrometer (EMXPlus, Bruker Corp., MA, USA) with an X-band cavity (ER 4103TM, Bruker Corp., MA, USA). Hyperfine coupling constants were obtained using the computer program Winsim (version 0.96; NIEHS, NIH, Research Triangle Park, NC, USA, https://www.niehs.nih.gov^40,44^. The following ESR parameters were used: microwave frequency of 9.8497 GHz, microwave power of 10 mW, sweep width of 5 mT around the center magnetic field of 351 mT, modulation frequency of 100 kHz, modulation amplitude of 0.2 mT, time constant of 164 ms, conversion time of 230 ms, and total scan time of 240 s.
