# Publisher Correction: Quantitative evaluation of SARS-CoV-2 inactivation using a deep ultraviolet light-emitting diode

**DOI:** 10.1038/s41598-021-94999-4

**Published:** 2021-08-04

**Authors:** Takeo Minamikawa, Takaaki Koma, Akihiro Suzuki, Takahiko Mizuno, Kentaro Nagamatsu, Hideki Arimochi, Koichiro Tsuchiya, Kaoru Matsuoka, Takeshi Yasui, Koji Yasutomo, Masako Nomaguchi

**Affiliations:** 1 Department of Post-LED Photonics Research, Institute of Post-LED Photonics, Tokushima University, 2-1 Minami-Josanjima, Tokushima, Tokushima 770-8506 Japan; 2 Department of Mechanical Science, Graduate School of Technology, Industrial and Social Sciences, Tokushima University, 2-1 Minami-Josanjima, Tokushima, Tokushima 770-8506 Japan; 3 PRESTO, Japan Science and Technology Agency (JST), 2-1 Minami-Josanjima, Tokushima, Tokushima 770-8506 Japan; 4 Research Cluster on “Multi-Scale Vibrational Microscopy for Comprehensive Diagnosis and Treatment of Cancer”, Tokushima University, 2-1 Minami-Josanjima, Tokushima, Tokushima 770-8506 Japan; 5 Department of Microbiology, Graduate School of Biomedical Sciences, Tokushima University, 3-18-15 Kuramoto, Tokushima, Tokushima 770-8503 Japan; 6 Department of Electrical and Electronic Engineering, Graduate School of Technology, Industrial and Social Sciences, Tokushima University, 2-1 Minami-Josanjima, Tokushima, Tokushima 770-8506 Japan; 7 Department of Immunology and Parasitology, Graduate School of Biomedical Sciences, Tokushima University, 3-18-15 Kuramoto, Tokushima, Tokushima 770-8503 Japan; 8 Department of Medical Pharmacology, Graduate School of Biomedical Sciences, Tokushima University, 3-18-15 Kuramoto, Tokushima, Tokushima 770-8503 Japan; 9 Department of Interdisciplinary Researches for Medicine and Photonics, Institute of Post-LED Photonics, Tokushima University, 3-18-15 Kuramoto, Tokushima, Tokushima 770-8503 Japan; 10 Research Cluster on “Immunological Diseases”, Tokushima University, 3-18-15 Kuramoto, Tokushima, Tokushima 770-8503 Japan

Correction to: *Scientific Reports* https://doi.org/10.1038/s41598-021-84592-0, published online 03 March 2021

The original version of this Article contained an error in Affiliation 1, which was incorrectly given as ‘Department of Electrical and Electronic Engineering, Graduate School of Technology, Industrial and Social Sciences, Tokushima University, 2-1 Minami-Josanjima, Tokushima, Tokushima 770-8506, Japan’. The correct affiliation is listed below:

‘Department of Post-LED Photonics Research, Institute of Post-LED Photonics, Tokushima University, 2-1 Minami-Josanjima, Tokushima, Tokushima 770-8506, Japan’

In addition, the affiliations for Kentaro Nagamatsu were incorrectly listed. The correct affiliations are listed below.

‘Department of Post-LED Photonics Research, Institute of Post-LED Photonics, Tokushima University, 2-1 Minami-Josanjima, Tokushima, Tokushima 770-8506, Japan’

‘Department of Electrical and Electronic Engineering, Graduate School of Technology, Industrial and Social Sciences, Tokushima University, 2-1 Minami-Josanjima, Tokushima, Tokushima, 770-8506, Japan’

The original Article has been corrected.

